# Acute Recanalization of Large Vessel Occlusion in the Anterior Circulation Stroke: Is Mechanical Thrombectomy Alone Better in Patients over 80 Years of Age? Findings from a Retrospective Observational Study

**DOI:** 10.3390/jcm10184266

**Published:** 2021-09-20

**Authors:** Dagmar Krajíčková, Antonín Krajina, Roman Herzig, Oldřich Vyšata, Libor Šimůnek, Martin Vališ

**Affiliations:** 1 Department of Neurology, Comprehensive Stroke Center, Charles University Faculty of Medicine and University Hospital, Sokolská 581, CZ-500 05 Hradec Králové, Czech Republic; dagmar.krajickova@fnhk.cz (D.K.); vysatao@gmail.com (O.V.); libor.simunek@email.cz (L.Š.); valismar@seznam.cz (M.V.); 2 Department of Radiology, Comprehensive Stroke Center, Charles University Faculty of Medicine and University Hospital, CZ-500 05 Hradec Králové, Czech Republic; antonin.krajina@fnhk.cz; 3 Department of Computing and Control Engineering, University of Chemistry and Technology in Prague, Czech Republic, CZ-166 28 Prague 6, Czech Republic

**Keywords:** anterior circulation, acute ischemic stroke, large vessel occlusion, elderly patients, mechanical thrombectomy, intravenous thrombolysis, bridging therapy, recanalization, clinical outcome

## Abstract

Real-world data report worse 3-month clinical outcomes in elderly patients with acute ischemic stroke (AIS) treated with mechanical thrombectomy (MT). The aim was to identify factors influencing clinical outcome in elderly patients with anterior circulation AIS treated with MT (±intravenous thrombolysis (IVT)). In a retrospective, monocentric study, analysis of prospectively collected data of 138 patients (≥80 years) was performed. IVT was an independent negative predictor (OR 0.356; 95% CI: 0.134–0.942) and female sex an independent positive predictor (OR 4.179, 95% CI: 1.300–13.438) of 3-month good clinical outcome (modified Rankin scale 0–2). Female sex was also an independent negative predictor of 3-month mortality (OR 0.244, 95% CI: 0.100–0.599). Other independent negative predictors of 3-month good clinical outcome were older age, lower pre-stroke self-sufficiency, more severe neurological deficit and longer procedural intervals. Mortality was also independently predicted by longer procedural interval and by the occurrence of symptomatic intracerebral hemorrhage (*p* < 0.05 in all cases). Our results demonstrated, that in patients aged ≥80 years with anterior circulation AIS undergoing MT (±IVT), IVT reduced the chance of 3-month good clinical outcome and female sex was associated with a greater likelihood of 3-month good clinical outcome and lower probability of 3-month mortality.

## 1. Introduction

Mechanical thrombectomy (MT) is currently the standard treatment for acute ischemic stroke (AIS) in the anterior circulation due to the emergent large vessel occlusion (ELVO). MT has been shown to lead to higher recanalization rates and better clinical outcomes in selected patients compared to intravenous thrombolysis (IVT) alone [1,2,3,4,5]. Although the guidelines recommend, unless there is a contraindication, treatment with intravenous recombinant tissue plasminogen activator (rtPA) before MT (bridging therapy) [6,7], there are doubts about the benefit of IVT preceding MT [8,9,10,11,12,13,14,15,16]. Real-world data consistently report worse 3-month clinical outcomes in elderly patients compared to younger patients [17,18,19,20,21,22,23,24]. Nevertheless, the factors influencing the clinical outcome in elderly patients have not been fully elucidated.

The aim was to identify factors influencing the recanalization rate, the occurrence of symptomatic intracerebral hemorrhage (sICH) and the clinical outcome in elderly patients (≥80 years) with anterior circulation AIS due to ELVO undergoing MT (±IVT) in routine clinical practice.

## 2. Materials and Methods

### 2.1. Data Source and Study Population

All relevant data used for this retrospective analysis were manually extracted from hospital information systems and available individual patient medical charts, including documentation from the referring hospital (in the case of patients with secondary transport), emergency physician notes, neurology notes, and medication administration records.

In a retrospective, monocentric study, a set of 138 consecutive elderly patients (≥80 years) with anterior circulation AIS due to ELVO treated with MT using second generation stent-retrievers (±IVT) from January 2013 to June 2019 in the Comprehensive Stroke Center (CSC), University Hospital in Hradec Králové, Czech Republic, was analyzed. Some patients were transported to the CSC directly, others were transported to the CSC for MT from primary stroke centers (PSC). The indication for MT was determined in accordance with the valid guidelines [6,25,26,27,28].

### 2.2. Computed Tomography Imaging

Non-enhanced computed tomography (NECT) of the brain with the assessment of the Alberta Stroke Program Early CT Score (ASPECTS) and CT angiography (CTA) of the cervical and intracranial arteries including the evaluation of the collateral system was performed in all patients. For CTA, a non-ionic contrast iomeprol (Iomeron 400, Bracco, Torviscosa, Italy) was administered intravenously (total volume of 60 mL, speed 4 mL/s). Patients treated more than 6 h from symptoms onset and patients with unknown AIS onset time also underwent perfusion CT (PCT) examination using 40 mL of the same contrast medium infused intravenously at 5 mL/s. The time to maximum (Tmax) delay > 6 s was used for the display of ischemic penumbra and, the relative cerebral blood flow < 30% of that in normal tissue for a diagnosis of ischemic core (irreversibly injured brain tissue) [29]. In patients with IVT administered in the PSC, a control brain NECT was performed in the CSC prior to MT initiation to exclude bleeding complications and the development of extensive cerebral infarction. A Somatom Definition AS + (Siemens, Forchheim, Germany) scanner was used for CT examinations.

### 2.3. Intravenous Thrombolysis

Intravenous thrombolysis with a standard dose of 0.9 mg/kg (maximum dose 90 mg) of recombinant tissue plasminogen activator (Actilyse, Boehringer Ingelheim, Ingelheim am Rhein, Germany) was applied in all patients with known stroke onset time fulfilling the inclusion and exclusion criteria according to the valid guidelines. Furthermore, 10% of the rtPA dose was administered as intravenous bolus, followed by a 60-min infusion of the remaining 90% of the dose [30].

### 2.4. Digital Subtraction Angiography and Mechanical Thrombectomy

Within a 6-h time window from AIS symptoms onset, MT was performed in patients with ASPECTS ≥ 6 on NECT. The result of PCT was crucial in patients treated more than 6 h from AIS symptoms onset and in patients with unknown AIS onset time—MT was performed in patients with a small ischemic core (≤70 mL; based of the results of previous studies [31]) and with the presence of ischemic penumbra. Patients without mismatch were excluded from therapy. A poor condition of the collateral system, if representing the only unfavorable prognostic factor, was not a reason for the exclusion from the MT. MT was started as soon as possible, not waiting for the IVT effect, if applied.

A biplane angio machine (Philips Allura FD 20/20, Best, The Netherlands) was used for DSA examination. Non-ionic contrast iodixanol (Visipaque 320, GE Healthcare AS, Oslo, Norway) was administered intra-arterially using a Seldinger technique (total volume of 6 mL, speed 7 mL/s). For MT, second-generation stent-retrievers were used—Trevo (Trevo ProVue, Concentric Medical, Mountain View, CA, USA), Solitaire (Solitaire, Covidien, Dublin, Ireland), Eric 4 (ERIC 4 Retrieval Device, MicroVention Terumo, Saint-Germain-en-Laye, France), and Preset (pREset, Phenox GmbH, Bochum, Germany). The choice of stent-retriever depended on the decision of the radiologist. In the majority of procedures, the balloon guiding catheter (Merci 8F, Concentric Medical, Mountain View, CA, USA) was placed within the internal carotid artery (ICA). In the presence of a loop, the catheter was placed below it. A microcatheter (0.021 inch) with stent-retriever was subsequently introduced. The stent-retriever was deployed across the occlusion and after 4 min the stent was slowly retrieved, while flow arrest in the accessing artery was applied by balloon inflation. Manual aspiration was applied through the guiding catheter via sidearm using a 20-cc syringe. In most cases, the procedure was performed under conscious sedation, without general anesthesia. Blood pressure was kept above 140 mm Hg.

### 2.5. Observed Parameters

The following data were collected: demographic data (age, sex, occurrence of vascular risk factors—arterial hypertension, diabetes mellitus, dyslipidemia, coronary artery disease, atrial fibrillation, prior stroke/transient ischemic attack), pre-intervention data (pre-stroke modified Rankin scale (mRS) value [32], direct/interhospital transfer, neurologic deficit—assessed using the National Institutes of Health Stroke Scale (NIHSS) [33], initial ASPECTS values, presence of leukoaraiosis (defined as CT signs of hypodense lesions around the anterior and posterior horns of the lateral ventricles [34]), location of vessel occlusion), and procedural characteristics (time intervals—the time when the patient was last seen normal was used in patients with an unknown AIS onset time, number of stent-retriever passes (SRPs), recanalization rate assessed using TICI score with successful recanalization defined as TICI 2b/3 [35], occurrence of sICH defined according to the Safe Implementation of Thrombolysis in Stroke-Monitoring Study (SITS-MOST) criteria [36] and 3-month clinical outcome assessed using mRS—with good clinical outcome defined as 0–2 points). Clinical outcome was assessed by a certified treating neurologist not blinded for the result of the MT. In patients, who were unable to attend a follow-up visit at the CSC, a telephone interview with another treating physician or the family members was used.

### 2.6. Statistical Analysis

The normality of the distributions was confirmed using the Kolmogorov–Smirnov test. Fisher’s exact test was used to assess the relationship between categorical variables. The Mann–Whitney U-test or unpaired *t*-test was used, depending on data normality, to evaluate the impact of quantitative parameters.

A multivariate linear logistic regression model was used for the prediction of successful recanalization, sICH occurrence, 3-month good clinical outcome, and 3-month mortality. Bonferroni test was used for multiple comparison. Multiple colinear time predictors have been reduced to “onset-to-groin puncture” and “groin puncture-to-end of intervention” independent predictors.

Standard multivariate logistic regression was separately used for each binary output variable of interest. In the initial model, 28 predictors were used. First, we examined possible hidden relationships between output variables and explanatory ones as they could cause difficulty to the estimation process on one hand, but on the other, could also provide direct implications between these two groups of variables.

Explanatory variables involved in such relationship with an output variable were excluded from the data set when estimating the corresponding model for this output variable. Then complete models were estimated using all input variables without hidden relationships with the dependent variable. As not all estimated coefficients in the complete models were significant at the level of 0.95, we examined the justification of their inclusion into the model with the likelihood ratio (LR) test. Due to the large number of explanatory insignificant variables and their possible mutual dependency in many cases, the testing was proceeded as follows. First, we removed all statistically insignificant variables from the complete model and used the LR test to verify whether such exclusion was statistically justifiable. If not, we returned the previously eliminated variables into the model in a piecewise mode according their *p* values in the full model until the null hypothesis of excluding the rest of them was not rejected by the LR test. For all explanatory variables included in final models we calculated their odds ratio and its corresponding confidence intervals.

The MATLAB Statistical Toolbox (MathWorks, Natick, MA, USA) was used for statistical analysis.

### 2.7. Ethics

The entire study was conducted in accordance with the Declaration of Helsinki of 1964 and its later amendments (including the last in 2013). All procedures were performed in accordance with institutional guidelines. The study was approved by the Ethics Committee of the University Hospital Hradec Králové (approval No. 201912 S16R). All conscious patients signed informed consent forms for the eligible and available diagnostics and treatment. Independent witnesses verified the signatures in cases in which there were technical problems.

## 3. Results

The set consisted of 138 elderly patients (≥80 years) (98 females; mean age 84.6 ± 3.5 years), out of whom 57 (41.3%) patients were treated with MT alone (MT-A) and 81 (58.7%) patients with MT + IVT. Baseline characteristics are presented in Table 1.

In patients treated with MT-A versus MT + IVT, there was significantly more frequent unknown symptom onset (38.6% versus 16.0%, *p* = 0.04) and ASPECTS values were significantly higher—both in the whole sets (mean 8.7 versus 8.1, *p* = 0.002) and in the subgroups of patients with direct transfer to the CSC (8.8 versus 8.1, *p* = 0.008).

As the first stent-retriever, Trevo was used 104 times (75.4%), Solitaire 32 times (23.2%), and both Eric 4 and Preset were used once. Two types of stent-retrievers were used twice during one procedure. One SRP was sufficient in 49.3% of patients, more than three SRPs were needed in 10.1% of patients. Furthermore, 26 out of 38 patients with secondary transport to the CSC were treated with IVT in the PSC. This represents only 32.1% out of 81 patients treated with IVT and this small proportion does not allow evaluation of the benefit and risk of this treatment prior to patients’ transfer to the CSC. As presented in Table 2, IVT administration did not delay the start of MT, the interval from the known time of onset-to-groin puncture was even slightly shorter in the IVT + MT group than in the MT-A group. Door intervention center-to-groin puncture time was shorter by about half in patients with secondary transport to the CSC than in patients with direct transfer, because the emergency department and intervention team were pre-activated by the PSC. In the whole group, sICH occurred in 6.5% of patients—more frequently in patients with unknown time of symptoms onset (8.6%), with interhospital transfer (7.9%), with leukoaraiosis (11.1%), and with IVT (8.6%). The same factors reduced the chances of the achievement of a good clinical outcome (30.4% in the whole group) and increased the mortality (36.2% in the whole group)—28.6% and 40.0% in patients with unknown time of symptoms onset, 26.3% and 42.1% with interhospital transfer, 22.2% and 42.6% with leukoaraiosis and, 25.9% and 40.7% with IVT. By day 10, only 26% of patients had died, with the largest number (40%) dying between days 31 and 90. Pre-stroke mRS affected both the achievement of good clinical outcome (45.5% in patients with pre-stroke mRS ≤ 2) and mortality (29.5% in patients with pre-stroke mRS ≤ 2 and 46.4% with pre-stroke mRS 3–5). Procedural characteristics and outcomes in particular groups are presented in Table 2.

As presented in Table 3, higher number of stent-retriever passes was identified as an independent negative predictor of the achievement of successful recanalization. Higher ASPECTS value was identified as an independent negative predictor and the presence of leukoaraiosis as an independent positive predictor of the sICH occurrence. IVT was identified as an independent negative predictor of the achievement of 3-month good clinical outcome. Female sex was identified as an independent positive predictor of the achievement of 3-month good clinical outcome and as an independent negative predictor of 3-month mortality. Other independent negative predictors of the achievement of 3-month good clinical outcome were, as expected, older age, lower pre-stroke self-sufficiency, more severe neurological deficit and longer time intervals (onset-to-groin, groin puncture-to-end of intervention). In addition to older age, mortality was independently predicted by the longer groin puncture-to-end of intervention interval and by the sICH occurrence (Table 3).

## 4. Discussion

About 30% of patients with AIS are aged 80 and over, 17% are even older than 85 years, and the proportion of elderly patients will continue to increase as the population ages [37,38]. The fact that AIS due to ELVO in elderly patients has a poor prognosis without MT (in the medical arm/IVT group of HERMES meta-analysis only 13.9% of these patients achieved mRS 0–2 and their mortality was 45.2% [18]) supports the legitimacy of the use of MT in this age group, although its results are significantly worse compared to younger patients despite similar successful recanalization rates [17,18,19,20,21,22,23,24]. In the HERMES meta-analysis, in patients treated with MT, mRS ≤ 2 was achieved in 46% of patients aged 57–77 years and only in 29.8% of patients aged ≥80 years, and, mortality was 15.3% and 28% in particular groups [1,3,5]. In the STRATIS registry of patients treated with MT, rates of good clinical outcomes significantly decreased for each 5-year increment of age from <65 to >90 years, from 64.3% to 26.5%, while mortality significantly increased from 7.9% to 35.1% [19]. In the TREVO registry, 33.7% of patients aged ≥80 years reached mRS ≤ 2 and their mortality was 27.2%, while the same values for younger patients were 62.2 and 9.6%, respectively [20]. Out of 560 patients treated with MT in seven US centers, 108 patients aged ≥80 years also had significantly worse clinical outcomes when compared with younger patients—mRS ≤ 2 was achieved in 20.5% versus 44.4% and mortality was 34.3% versus 20% in particular groups [21]. Our results are comparable to those published—3-month good clinical outcome (mRS ≤ 2) was achieved in 30.4% of our patients aged ≥80 years and their 3-month mortality was 36.2%.

Older patients have poor clinical outcome at 3 months despite high rates of the achieved recanalization. In our group, successful recanalization (TICI 2b/3) was achieved in 76.1% of patients aged ≥80 years. In the work published in 2016, early neurological improvement depended on successful recanalization, not on the age of MT-treated patients. However, older patients exhibited poor mid-term functional outcome due to post-stroke complications and other factors that were not, or only indirectly, related to the brain tissue damage induced by the incident stroke [39]. Corresponding to our finding is that a larger proportion of patients aged ≥80 years died later—40% of them between days 31 and 90, when a direct association with brain tissue damage due to stroke is unlikely. Factors that adversely affect the prognosis in older patients may be pre-existing neuronal loss and reduced neuronal plasticity, leukoaraiosis, higher rate of comorbidities, and post-stroke complications (e.g., infections or injuries due to falls) [40,41]. In our patients with leukoaraiosis, when compared to the whole group, we recorded a higher occurrence of sICHs (11.1% versus 6.5%) and higher 3-month mortality (42.6% versus 36.2%) and a less frequent 3-month good clinical outcome (22.2% versus 30.4%). Due to pre-existing changes, the brain of elderly patients has depleted or borderline functional capacity, and is therefore able to tolerate only a small volume of infarction [42,43].

Whether treatment with IVT before MT in patients with anterior circulation stroke due to ELVO is of any benefit is currently one of the most important unanswered questions in acute stroke management [16]. To date, the results of randomized control trials (RCTs), as well as several registries and multicenter and single-center studies, suggest that MT-A is equally effective and not inferior to MT + IVT, if patients are immediately treated in stroke centers with rapid access to endovascular procedures. This was recently confirmed by multicenter RCTs from China and Japan (DIRECT-MT [44], DEVT [45], and SKIP [46]), which did not show a statistically significant difference between patients treated with MT-A versus MT + IVT in self-sufficiency at 3 months (36.4%, 54.3%, and 59.4% versus 36.8%, 46.6%, and 57.3%, resp.) or in mortality (17.7%, 17.2%, and 7.9% versus 18.8%, 17.8%, and 8.7%, resp.). In the DIRECT-MT trial, even a 5% higher successful recanalization rate achieved in patients treated with MT + IVT did not lead to a better functional outcome, and a relatively lower achievement of independence in both treatment groups was probably due to remarkably low rates of favorable collaterals [44]. Meta-analysis of six older RCTs with 940 patients treated with IVT and endovascular treatment and 107 patients treated with endovascular treatment alone showed that endovascular treatment was associated with a higher likelihood of the achievement of better outcome at 3 months—both in the group with IVT (OR 1.83) and without IVT (OR 2.47) [10]. The results of the meta-analysis of eight RCTs were similar [13]. No statistically significant differences were also confirmed by the pooled analysis of SWIFT and STAR studies [11]. Similar clinical outcomes at 90 days (either good clinical outcome, mortality, or both) in patients treated with MT-A and MT + IVT were also found in the Catalan SONIIA registry from 2011 to 2015 [12], Bernese Stroke Registry (with the exception of a lower mortality in the MT-A group) [9] (even after adding patients from the Essen registry, despite the fact that MT was delayed by 1 h in patients with MT + IVT [14]), and in other studies [8,15]. However, there are also works reporting better outcomes in patients treated with MT + IVT than with MT-A. In the German REVASK registry from 2012 to 2013, comparing patients with MT + IVT and MT-A, good 3-month clinical outcome was achieved in 47.5% versus 38.1% and mortality was 17.8% versus 27.8% in particular groups. As well as in our patient set, patients with MT + IVT had shorter times to groin puncture [47]. IVT was a predictor of the achievement of good outcome in another German registry from 2015 to 2018 (OR 1.49) [23], and better functional outcome (adjusted OR (aOR) 1.47 and 1.4, resp.) and lower mortality (aOR 0.58 and 0.74, resp.) were found in patients treated with IVT + MT versus MT-A in the Dutch MR CLEAN registry from 2014 to 2016 [48] and in the international SITS-ISTR registry from 2014 to 2016 [49]. Data regarding the effect of IVT preceding MT on ICH frequency vary. In the SONIIA registry [12], MR CLEAN registry [48], and SITS-ISTR registry [49], the occurrence of sICH in both treatment groups was similar—3.4%, 5.9%, and 3.5%, respectively, in the IVT + MT group versus 2.7%, 5.3%, and 3.0%, respectively, in the MT-A group. On the other hand, the German registries showed a significantly higher incidence of sICH in patients with MT + IVT (4.5%, 5.3%, and 3.5%, resp.) versus patients with MT-A (2.5%, 2.7%, and 1.6%, resp.) [9,14,47] and also of ICH according to the ECASS II definition [50] irrespective of the new clinical symptoms (15% versus 11% in particular groups) [23]. In the Bernese Stroke Registry, lower rate of asymptomatic ICH was observed in the MT-A versus MT + IVT group (12.5% versus 20%) [9]. Some authors [51,52] state that extensive leukoaraiosis may increase the risk of sICH in addition to IVT, and our results correspond with this.

We found the evaluation of the effect of IVT adjusted for age only in the study from Australia from 2016 to 2018 performed by Sharobeam et al. [22]. In older (≥80 years) versus younger patients, treated for anterior circulation stroke with MT using second generation stent-retrievers, IVT was used in 34% versus 45%, procedure duration was 35 versus 31 min, the occurrence of sICH according to the SITS-MOST definition was 4% versus 5%, mRS 0–2 at 90 days was achieved in 28% versus 55%, and mortality rate was 27% versus 16% in particular groups. IVT was significantly associated with a favorable outcome in younger patients (OR 2.90), but not in elderly patients. In our study, IVT was identified as an independent negative predictor of the achievement of 3-month good clinical outcome (OR 0.356).

Females, representing 71% of our patients, achieved a better outcome—female sex was identified as an independent positive predictor of the achievement of 3-month good clinical outcome (OR 4.179) and as an independent negative predictor of 3-month mortality (OR 0.244). The conclusions of recent studies that reported sex differences in clinical outcome after MT are inconsistent. Studies analyzing large groups of younger patients (median age of females 69–70 years) from RCTs conclude that despite the fact that females were older and had a higher comorbidity compared to males, their clinical outcome after MT was comparable and according to the study performed by Sheth et al., females had significantly more years of optimal life (disability-adjusted life year) when compared to males (10.6 versus 8.5 years) [53,54]. In contrast, in the Japanese RESCUE registry, females (mean age 79.7 years), when compared to males (mean age 72.8 years), were less likely to achieve a good clinical outcome (27.3% versus 44.2%; aOR 0.80) and had higher mortality (12.3% versus 9.9%; aOR 0.78) [55]. The most similar to our cohort is the cohort of 279 patients treated with MT in a single, large, academic CSC in the USA between 2015 and 2017. Females, representing 52% of patients, were older than males (median age 81 versus 71.5 years). Although at discharge both males and females had a similar probability of functional independence (aOR 0.71), at 90 days the probability of functional independence was apparently lower in females (aOR 0.37). Post hoc multivariate logistic regression analysis in subgroups by age indicated a greater sex difference for independence in females under 75 years (OR 0.24), while OR for females older than 75 years compared to males was 0.54. However, the authors point out that the results of the subanalysis may not be reliable and are likely underpowered. The fact that both rates of successful recanalization and clinical outcome at discharge were similar between females and males, suggests that a worse long-term clinical outcome may reflect a less favorable situation of females not directly related to MT, such as their more frequent social isolation or lower social level. Females in this group were less likely to be married (38.2% versus 67.5%) and were more likely to have government-sponsored insurance (86.8% versus 73.1%) when compared to males [56]. The absence of greater social disparities and the general availability of outpatient or inpatient social services for the elderly in our country may have contributed to better long-term clinical outcomes after MT in females in our cohort, but do not explain the difference in outcome between the sexes.

Other independent negative predictors of the achievement of 3-month good clinical outcome were, as expected, older age, lower pre-stroke self-sufficiency, more severe neurological deficit, and longer time intervals (onset-to-groin, groin puncture-to-end of intervention). In addition to older age, mortality was independently predicted by the longer groin puncture-to-end of intervention interval and by the sICH occurrence.

Several limitations of the present study should be mentioned. First, it has a retrospective character with a sample extracted from a single stroke center database; therefore, the results may not be generalizable. Second, the data collection methods among databases may be the source of selection bias. Third, a relatively small number of patients in particular treatment subgroups limits the power for comparative analysis.

## 5. Conclusions

Our results demonstrated, that in patients with anterior circulation AIS due to ELVO aged ≥ 80 years and undergoing MT (±IVT), IVT reduced the chance of the achievement of 3-month good clinical outcome and female sex was associated with a greater likelihood of the achievement of 3-month good clinical outcome and lower probability of 3-month mortality. However, these results require verification on a larger set of patients.

## Figures and Tables

**Table 1:** Baseline characteristics.

Characteristic	MT-A (*n* = 57)	IVT + MT (*n* = 81)	*p*
Age, (years) ^†^	84.6 ± 3.7 (83) (82–87)	84.2 ± 3.4 (84) (81.75–87)	0.51
Female sex	43 (75.4)	55 (67.9)	0.79
Medical history			
Arterial hypertension	50 (87.7)	72 (88.9)	1.00
Diabetes mellitus	19 (33.3)	25 (30.9)	0.86
Dyslipidemia	21 (36.8)	30 (37.0)	1.00
Coronary artery disease	22 (38.6)	27 (33.3)	0.74
Atrial fibrillation	22 (38.6)	34 (42.0)	0.87
Prior stroke/TIA	15 (26.3)	17 (21.0)	0.69
Pre-stroke mRS			
0–2	32 (56.1)	56 (69.1)	0.49
3–5	15 (26.3)	13 (16.0)	0.30
Uncertain	10 (17.5)	12 (14.8)	0.82
Unknown symptom onset	22 (38.6)	13 (16.0)	0.04
Direct transfer to the CSC	45 (78.9)	55 (67.9)	0.18
Initial qualifying NIHSS ^†^	14.9 ± 4.6 (15) (12–18)	15.0 ± 4.9 (15) (13–17)	0.87
Initial CT characteristics			
ASPECTS—all patients ^†^	8.7 ± 1.0 (9) (8–9)	8.1 ± 1.4 (8) (7–9)	0.002
ASPECTS—direct transfer to the CSC ^†^	8.8 ± 1.1 (9) (9–10)	8.1 ± 1.4 (8) (7–9)	0.008
ASPECTS—interhospital transfer ^†^	8.4 ± 0.8 (9) (8–9)	7.9 ± 1.3 (8) (7–9)	0.24
Leukoaraiosis	21 (36.8)	33 (40.7)	0.87
Occluded vessel			
M1	35 (61.4)	47 (58.0)	0.89
M2	10 (17.5)	11 (13.6)	0.64
Distal ICA + M1 (L occlusion)	9 (15.8)	11 (13.6)	0.63
Distal ICA + M1 + A1 (T occlusion)	1 (1.8)	4 (4.9)	0.65
ICA + M1 (tandem occlusion)	2 (3.5)	8 (9.9)	0.09
IVT	0 (0)	81 (100.0)	
IVT ≤ 3 h	0 (0)	75 (92.6)	

Data are *n* (%) for categorical variables or mean ± SD (median) (IQR) for numerical variables ^†^. ASPECTS, Alberta Stroke Program Early CT Score; CSC, Comprehensive Stroke Center; CT, computed tomography; ICA, internal carotid artery; IQR, interquartile range; IVT, intravenous thrombolysis; M1, middle cerebral artery—segment 1; M2, middle cerebral artery—segment 2; mRS, modified Rankin scale; MT, mechanical thrombectomy; MT-A, MT alone; *n*, number of patients; NIHSS, National Institutes of Health Stroke Scale; SD, standard deviation; TIA, transient ischemic attack.

**Table 2:** Procedural characteristics and outcomes.

Characteristic		MT-A (*n* = 57)	IVT + MT (*n* = 81)	*p*
Time intervals (min)	Onset-to-needle ^†^		117.7 ± 38.6 (120.0) (98.5–140.0)	
	Onset-to-groin puncture ^†^	209.0 ± 93.4 (184.0) (141–265)	197.0 ± 63.3 (178.5) (145–229.5)	0.42
	Door intervention center-to-groin puncture ^†^	78.6 ± 32.9 (79.5) (56–106)	72.8 ± 27.8 (74) (51.75–91.25)	0.31
	Direct transfer to the CSC ^†^	89.5 ± 27.7 (85.5) (70.5–105)	85.5 ± 22.0 (83.0) (72.5–101)	0.42
	Interhospital transfer ^†^	40.8 ± 22.2 (30.0) (27–50.5)	47.5 ± 19.2 (40.5) (32.5–56)	0.35
	Groin puncture-to-end of intervention ^†^	46.8 ± 27.2 (40.0) (28–58)	46.2 ± 27.3 (38.5) (25–56)	0.91
	Onset-to-end of intervention ^†^	257.7 ± 98.2 (255) (173–313)	242.8 ± 67.3 (228.5) (191.5–286)	0.37
Stent-retriever passes	All patients ^†^	2.1 ± 1.6 (2) (1–3)	1.9 ± 1.0 (2) (1–3)	
	1	27 (47.4)	41 (50.6)	0.88
	2	14 (24.6)	19 (23.5)	1.00
	≥3	7 (12.3)	7 (8.7)	0.58
Recanalization grade	TICI 0	6 (10.5)	2 (2.5)	0.08
	TICI 1	0 (0)	2 (2.5)	0.51
	TICI 2a	9 (15.8)	14 (17.3)	1.00
	TICI 2b	14 (24.6)	25 (30.9)	0.58
	TICI 3	28 (49.1)	38 (46.9)	0.88
	TICI ≥2b	42 (73.7)	63 (77.8)	0.90
sICH occurrence	All patients	2 (3.5)	7 (8.6)	0.32
	Pre-stroke mRS ≤ 2	1/32 (3.1)	5/56 (8.9)	0.41
	Unknown symptom onset	1/22 (4.5)	2/13 (15.4)	0.55
	Direct transfer to the CSC	2/45 (4.4)	4/55 (7.3)	0.69
	Interhospital transfer	0 (0)	3/26 (11.5)	
	Leukoaraiosis	1/21(4.8)	5/33 (15.2)	0.40
	IVT		7 (8.6)	
3-month good clinical outcome (mRS 0–2)	All patients	21 (36.8)	21 (25.9)	0.38
	Pre-stroke mRS ≤ 2	21/32 (65.7)	19/56 (33.9)	0.12
	Unknown symptom onset	8/22 (36.4)	2/13 (15.4)	0.46
	Direct transfer to the CSC	17/45 (37.8)	15/55 (27.3)	0.54
	Interhospital transfer	4/12 (33.3)	6/26 (23.1)	0.71
	Leukoaraiosis	5/21 (23.8)	7/33 (21.2)	1.00
	IVT		21 (25.9)	
3-month mortality (mRS 6)	All patients	17 (29.8)	33 (40.7)	0.21
	Day 1–10	5/17 (29.4)	8/33 (24.2)	0.76
	Day 11–30	6/17 (35.3)	11/33 (33.3)	1.00
	Day 31–90	6/17 (35.3)	14/33 (42.4)	1.00
	Pre-stroke mRS ≤ 2	7 (21.9)	19 (33.9)	0.48
	Unknown symptom onset	8/22 (36.4)	6/13 (46.2)	0.75
	Direct transfer to the CSC	12/45 (26.7)	22/55 (40.0)	0.42
	Interhospital transfer	5/12 (41.7)	11/26 (42.3)	1.00
	Leukoaraiosis	8/21 (38.1)	15/33 (45.5)	0.80
	IVT		33 (40.7)	

Data are *n* (%) for categorical variables or mean ± SD (median) (IQR) for numerical variables ^†^. CSC, Comprehensive Stroke Center; IQR, interquartile range; IVT, intravenous thrombolysis; mRS, modified Rankin scale; MT, mechanical thrombectomy; MT-A, MT alone; *n*, number of patients; SD, standard deviation; sICH, symptomatic intracerebral hemorrhage; TICI, Thrombolysis in Cerebral Infarction.

**Table 3:** Significant independent predictors of the observed parameters—results of multivariate logistic regression analysis.

Observed Parameter	Predictor	OR (95% CI)	*p*
Successful recanalization (TICI 2b/3)	Number of stent-retriever passes	0.537 (0.376–0.766)	6 × 10^−4^
sICH	ASPECTS	0.559 (0.320–0.977)	0.041
	Leukoaraiosis	4.947 (1.036–23.619)	0.045
3-month good clinical outcome (mRS 0–2)	Age	0.803 (0.689–0.937)	0.005
	Female sex	4.179 (1.300–13.438)	0.016
	Pre-stroke mRS	0.052 (0.006–0.477)	0.009
	NIHSS value	0.856 (0.756–0.968)	0.013
	IVT	0.356 (0.134–0.942)	0.038
	Onset-to-groin interval	0.991 (0.984–0.999)	0.023
	Groin puncture-to-end of intervention	0.964 (0.941–0.987)	0.002
3-month mortality (mRS 6)	Age	1.250 (1.106–1.413)	3 × 10^−4^
	Female sex	0.244 (0.100–0.599)	0.002
	Groin puncture-to-end of intervention	1.015 (1.001–1.030)	0.040
	sICH	6.681 (1.171–38.105)	0.032

ASPECTS, Alberta Stroke Program Early CT Score; CI, confidence interval; IVT, intravenous thrombolysis; mRS, modified Rankin scale; NIHSS, National Institutes of Health Stroke Scale; OR, odds ratio; sICH, symptomatic intracerebral hemorrhage; TICI, Thrombolysis in Cerebral Infarction.

## Data Availability

The datasets analyzed during the current study are available from the corresponding author on reasonable request.
